# Sensitivity to systemic therapy for metastatic breast cancer in *CHEK2* 1100delC mutation carriers

**DOI:** 10.1007/s00432-015-1981-7

**Published:** 2015-05-10

**Authors:** Mieke Kriege, Agnes Jager, Antoinette Hollestelle, Els M. J. J. Berns, Jannet Blom, Marion E. Meijer-van Gelder, Anieta M. Sieuwerts, Ans van den Ouweland, J. Margriet Collée, Judith R. Kroep, John W. M. Martens, Maartje J. Hooning, Caroline Seynaeve

**Affiliations:** 1 Department of Medical Oncology, Erasmus MC Cancer Institute, Groene Hilledijk 301, 3075 EA Rotterdam, The Netherlands; 2 Departments of Clinical Genetics, Erasmus MC Cancer Institute, Rotterdam, The Netherlands; 3 Department of Clinical Oncology, Leiden University Medical Centre, Leiden, The Netherlands

**Keywords:** Metastatic breast cancer, *CHEK2* 1100delC, Chemotherapy, Endocrine therapy, Response, Survival

## Abstract

**Purpose:**

The role of CHEK2 in DNA repair by homologous recombination suggests that *CHEK2*-associated breast cancer (BC) patients might be more sensitive to chemotherapy inducing double-strand DNA breaks, but results hereon are lacking. We compared the sensitivity to first-line chemotherapy and endocrine therapy between *CHEK2* 1100delC and non-*CHEK2* metastatic breast cancer (MBC) patients.

**Methods:**

Sixty-two *CHEK2* 1100delC MBC patients were selected from three cohorts genotyped for *CHEK2* 1100delC (one non-*BRCA1/2* cohort and two sporadic cohorts). Controls were 62 non-*CHEK2* MBC patients, matched for age at and year of primary BC diagnosis, and year of metastatic disease. Objective response rate (complete and partial response) to, and progression-free survival (PFS) and overall survival (OS) after start of first-line chemotherapy and endocrine therapy were compared between *CHEK2* and non-*CHEK2* patients.

**Results:**

Median age at BC diagnosis was 46 and 51 years at MBC diagnosis. First-line chemotherapy consisted of anthracycline-based chemotherapy (*n* = 73), taxanes (*n* = 16), CMF(-like) chemotherapy (*n* = 33) and taxane/anthracycline regimens (*n* = 2). *CHEK2* and non-*CHEK2* patients had a comparable objective response rate (44 vs. 52 %). Also, PFS and OS after start of chemotherapy were comparable between both patient groups (hazard ratio 0.91; 95 % confidence interval 0.63–1.30 and 1.03; 95 % CI 0.71–1.49, respectively). Thirty-six *CHEK2* and 32 non-*CHEK2* patients received first-line endocrine therapy (mainly tamoxifen) for MBC. No significant differences were observed in objective response rate to, and PFS and OS after start of endocrine therapy.

**Conclusion:**

No differential efficacy of chemotherapy and endocrine therapy given for MBC was observed in *CHEK2* versus non-*CHEK2* patients.

## Introduction

*CHEK2* is a tumor suppressor gene associated with a moderately increased cumulative lifetime breast cancer (BC) risk (1.4- to 3-fold; Cybulski et al. 2011; Meijers-Heijboer et al. 2002; Weischer et al. 2008). The *CHEK2* gene encodes for the protein kinase CHEK2 which plays a critical role in cell cycle control and DNA damage repair. In response to double-strand DNA breaks, CHEK2 is activated by ataxia telangiectasia mutated (ATM) and is involved in cell cycle control, DNA repair and apoptosis. CHEK2 kinase phosphorylates TP53 and BRCA1, whereupon BRCA1 represses the non-homologous end-joining pathway and activates the homologous recombination repair pathway (Nevanlinna and Bartek 2006; Roeb et al. 2012; Tung and Silver 2011). Different *CHEK2* variants have been described, and in the Netherlands, the prevalence of the *CHEK2* 1100delC variant is relatively high, while other *CHEK2* variants (IVS2 + 1G > A, del5395, 1157T) are very rare (The CHEK2 Breast Cancer Case Control Consortium 2004; Hollestelle et al. 2010; Meijers-Heijboer et al. 2002).

BC patients carrying the germline *CHEK2* 1100delC mutation mainly develop estrogen receptor (ER)-positive BC (de Bock et al. 2006; Nagel et al. 2012; Weischer et al. 2012) and have an twofold to threefold increased risk of developing contralateral BC. Moreover, a worse distant disease-free survival and overall survival (OS) for *CHEK2* 1100delC BC patients have been reported (de Bock et al. 2004; Kriege et al. 2014; Schmidt et al. 2007; Weischer et al. 2012). A plausible explanation for the worse survival might be a decreased sensitivity to (adjuvant) systemic therapy in *CHEK2* compared to non-*CHEK2* BC patients. On the other hand, the function of the CHEK2 protein kinase in the repair of double-strand DNA breaks suggests that BC patients carrying a *CHEK2* mutation might have an increased sensitivity to chemotherapeutic agents causing double-strand DNA breaks, such as platinum, alkylating agents and/or anthracyclines (Nevanlinna and Bartek 2006). However, data on the efficacy of systemic therapy in *CHEK2*-associated BC patients are very limited so far. In a previous study of our group regarding distant disease-free survival and breast cancer-specific survival of primary BC, no differential effect of adjuvant chemotherapy or endocrine therapy was observed in *CHEK2* 1100delC compared with non-*CHEK2* breast cancer patients (Kriege et al. 2014).

To further elucidate the potentially differential efficacy of systemic therapy among *CHEK2* mutation carriers compared to non-*CHEK2* patients, we assessed the efficacy of standard first-line chemotherapy given for metastatic breast cancer (MBC) in *CHEK2* 1100delC compared with non-*CHEK2* patients. Furthermore, the efficacy of first-line endocrine therapy for MBC was also determined in both groups.

## Patients and methods

For the current study, we used a database available from a previous study (Kriege et al. 2014), consisting of women with invasive BC genotyped for *CHEK2* 1100delC from three different cohorts. One cohort consisted of non-*BRCA1/2* patients from the Rotterdam Family Cancer Clinic (non-*BRCA1/2* cohort). The two other cohorts consisted of sporadic BC patients from (1) the ORIGO study, a study designed to investigate the prevalence of *BRCA1/2* mutations in an unselected BC population (ORIGO cohort) and (2) the Rotterdam Medical Oncology Tumor Bank database (RMOT cohort). More details regarding these cohorts have previously been described (Kriege et al. 2014). The inclusion criteria for the previous study were first BC diagnosed before age 80 and after 1970, and follow-up data available. Patients with a proven *BRCA1* or *BRCA2* mutation were excluded. For the current study, BC patients with a *CHEK2* 1100delC mutation and treated with first-line chemotherapy (irrespective of type) for metastatic disease were selected from the database.

The *CHEK2* 1100delC mutation status of the respective BC patients in the three cohorts was determined by either allele-specific oligonucleotide hybridization or Taqman genotyping as described earlier. DNA was isolated from peripheral blood of patients from the non-*BRCA1/2* and ORIGO cohorts and from freshly frozen tumor tissue of patients from the RMOT cohort as described (Kriege et al. 2014). In this paper, we refer to *CHEK2* 1100delC mutation carriers as *CHEK2* mutation carriers.

**Fig. 1:**
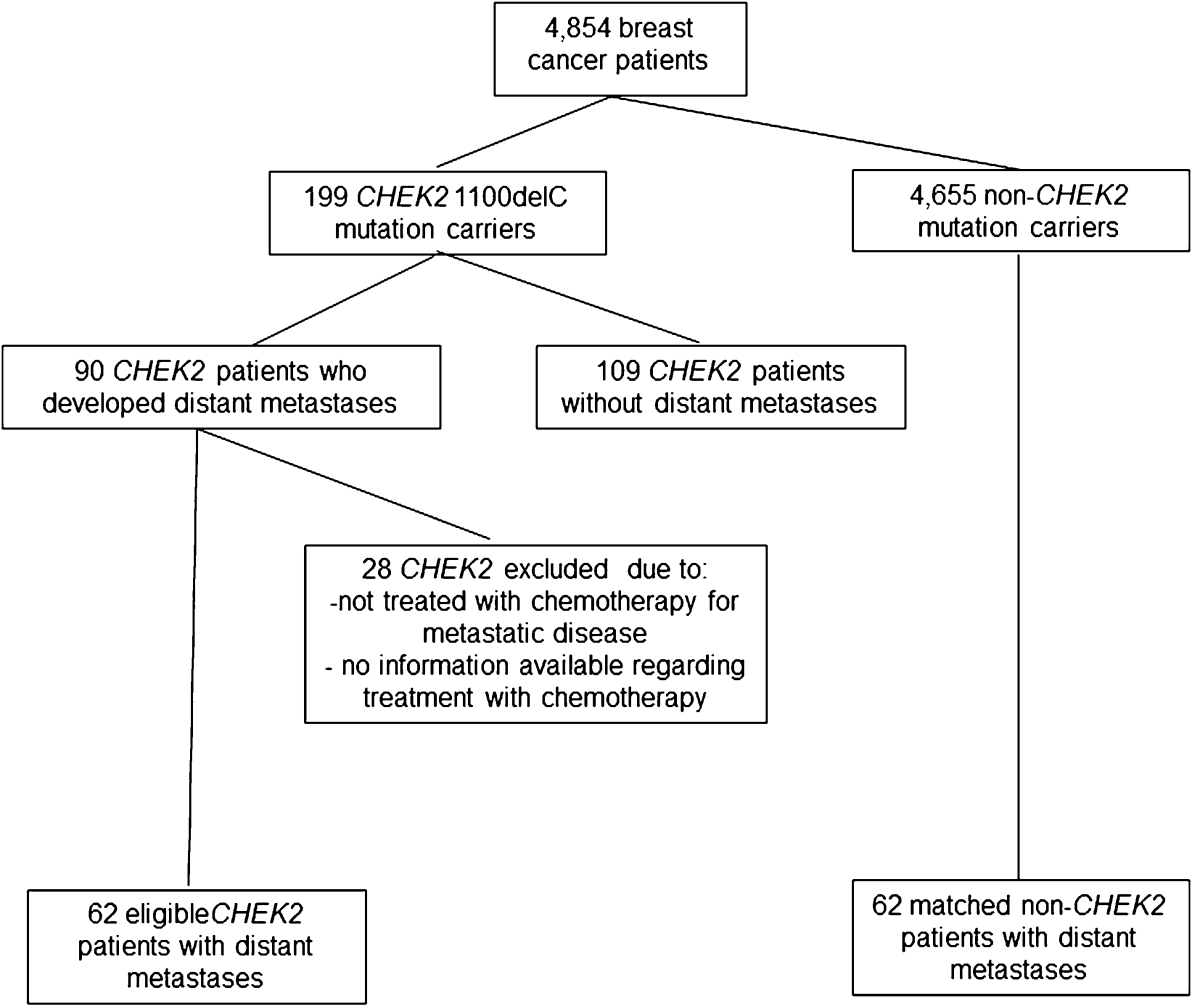
Flowchart for patient selection

In total, 4854 patients were tested for *CHEK2* 1100delC. From this cohort, 199 (4.1 %) had a *CHEK2* 1100delC mutation of whom 90 (45.2 %) patients developed distant metastases (Fig. 1). From the 90 *CHEK2* 1100delC BC patients with distant metastases, 28 patients were excluded because they were not treated with chemotherapy for MBC (*n* = 22) or because information on treatment for MBC was incomplete (*n* = 6). The eventually 62 eligible *CHEK2* mutation carriers with MBC were matched (1:1) for age at and period of primary BC diagnosis (within 5 years) and year of diagnosis of metastatic disease (within 5 years) with non-*CHEK2* patients treated with chemotherapy for MBC, selected from the same database.

This study was approved by the Medical Ethical Committee of the Erasmus MC, Rotterdam, the Netherlands (MEC 2009-344).

### Data collection

For all *CHEK2* and non-*CHEK2* MBC patients included in the current study (*n* = 124), data were extracted from hospital charts concerning patient and tumor characteristics, (systemic) treatment for primary BC, and if applicable for contralateral BC and loco–regional recurrence, date and location of distant metastases, type of and response to treatment for metastatic disease, date of progressive disease during or after first-line therapy (chemotherapy and/or endocrine therapy) for MBC and death. End date of the study was date of death, date of last medical contact or date of end of study (1-6-2013), whichever came first.

### Endpoints

The endpoints in this study were objective response rate and clinical benefit rate to, and progression-free survival (PFS) and OS after start of first-line chemotherapy and first-line endocrine therapy for MBC. Objective response was defined as complete response (CR) or partial response (PR), and clinical benefit as objective response or stable disease >6 months, according to International Union Against Cancer Criteria (Hayward et al. 1977), as a large part of the patient was treated before the introduction of the RECIST criteria in 2000. PFS after first-line systemic therapy (chemotherapy or endocrine therapy) was defined as time between start of either chemotherapy or endocrine therapy and date of progressive disease. OS after first-line systemic therapy (chemotherapy or endocrine therapy) was defined as time between start of either chemotherapy or endocrine therapy and death due to any reason [one patient (with a CHEK2 mutation) died from another reason than breast cancer (heart disease)].

For the study aims regarding the efficacy of first-line chemotherapy, all patients who received chemotherapy for MBC were included, irrespective of whether they also were treated with endocrine therapy for MBC before chemotherapy. Patients who were treated with an anthracycline-based regimen (without taxanes) as first-line chemotherapy were analyzed separately. For the analyses regarding the efficacy of endocrine therapy for MBC, we only included patients receiving endocrine therapy before chemotherapy (*n* = 68; 36 *CHEK2* and 32 non-*CHEK2* patients), because endocrine therapy after chemotherapy was most times given as consolidation therapy.

### Statistical analyses

Differences in patient, tumor and treatment characteristics between *CHEK2* and non-*CHEK2* patients were tested by a Chi-square test or Fisher’s exact test for categorical variables or by a *t* test for continuous variables. Differences in response rate to first-line chemotherapy and to first-line endocrine therapy between the patient groups were tested by a Chi-square test with linear by linear association. The Kaplan–Meier method was used to calculate PFS and OS after start of first-line chemotherapy and first-line endocrine therapy. Censoring events were date of last medical contact or end date of the study. Differences in PFS and OS between *CHEK2* and non-*CHEK2* patients were tested by a log-rank test. The Cox proportional hazard method was used to calculate univariate and multivariate hazard ratios (HR) and 95 % confidence intervals (CI) for the risk of progression and death after start of first-line chemotherapy and after start of first-line endocrine therapy for MBC in *CHEK2* versus non-*CHEK2* patients. Potential confounders included in the multivariate model were as follows: age at diagnosis (<50; ≥50), ER status (negative; positive; unknown), adjuvant chemotherapy (yes; no), metachronous contralateral BC (yes; no), type of first distant metastases (soft tissue; bone; visceral) and distant disease-free interval (<2 years; ≥2 years) and for the models regarding chemotherapy also endocrine therapy for MBC before chemotherapy (yes; no) and time between first distant metastasis and start of first-line chemotherapy (continuous).

Two-sided *P* values <0.05 were considered statistically significant. All analyses were performed with the use of SPSS software (version 21.0).

## Results

### Patient, tumor and treatment characteristics

Patient, tumor and adjuvant treatment characteristics of *CHEK2* 1100delC and non-*CHEK2* BC patients are depicted in Table 1. The median age at primary BC diagnosis was 45.4 years for the *CHEK2* mutation carriers and 46.7 years for the non-*CHEK2* patients, while the median age at the diagnosis of first distant metastases was 51.0 and 52.2 years, respectively. No significant differences in these tumor characteristics were observed between *CHEK2* mutation carriers and non-*CHEK2* patients. Tumors were mainly ER positive in both groups (89 and 75 % in *CHEK2* and non-*CHEK2* BC patients, respectively). Data regarding human epidermal growth factor receptor 2 (HER2) status are not mentioned, as these were mostly missing. First distant metastases occurred in bone for 47 % of the mutation carriers and 44 % of the non-*CHEK2* patients, respectively, and in viscera for 50 and 48 % of the patients, respectively.

**Table 1:** Patient, tumor and adjuvant treatment characteristics

	*CHEK2*		Non-*CHEK2*		*P*
	*N*	%	*N*	%	
Number of patients	62		62		
Median age at diagnosis (years)	45.4		46.7		0.76
Range	25.5–67.1		24.0–67.5		
Median age at diagnosis M1 (years)	51.0		52.2		0.69
Range	30.0–69.2		27.7–68.7		
Year of diagnosis M1					
<1990	8	13	7	11	0.43
1990–2000	30	48	26	42	
≥2000	24	39	29	47	
Tumor size					
T1	24	41	13	22	0.05
T2	26	44	32	56	
T3, T4	9	15	13	22	
Unknown	3		4		
Node positive	35	57	40	67	0.29
Not done/unknown	1		2		
M1 at diagnosis	3	5	2	3	1.00
Histologic grade					
I	8	18	5	10	0.33
II	13	29	15	30	
III	24	53	30	60	
Unknown	17		12		
Estrogen receptor positive	54	89	46	75	0.06
Unknown	1		1		
Progesterone receptor positive	42	79	36	67	0.14
Adjuvant chemotherapy*					
No	32	54	29	48	0.81
Anthracyclines	17	29	19	32	
Other	10	17	12	20	
Not applicable (M1)	3		2		
Adjuvant endocrine therapy^a^					
No	40	69	38	63	0.56
Yes	18	31	22	37	
Not applicable (M1) or unknown	4		2		
Contralateral breast cancer					
Yes	12	19	5	8	0.07
No	50	81	57	92	
Distant disease-free interval					
≤1 year	7	11	6	10	0.93
1–2 years	11	18	13	21	
2–5 years	21	34	21	34	
>5 years	23	37	22	35	
Site of first distant metastasis^b^					
Soft tissue	2	3	5	8	0.50
Bone	29	47	27	44	
Visceral	31	50	30	48	

^a^Including chemotherapy or endocrine therapy for a second breast cancer or for a loco–regional recurrence

^b^In case of multiple sites, the site with the worst prognosis was chosen (visceral > bone > soft tissue)

### First-line chemotherapy

Type of and response to first-line chemotherapy for MBC are shown in Table 2. First-line chemotherapy for MBC mainly consisted of anthracycline-based chemotherapy, being given to 34 (55 %) *CHEK2* and 39 (63 %) non-*CHEK2* patients, of whom 11 patients (six *CHEK2* and five non-*CHEK2*) continued with CMF after the maximal cumulative anthracycline dose (data not shown). The number of patients receiving endocrine therapy after first-line chemotherapy (as consolidation therapy) was comparable for the *CHEK2* mutation carriers (*n* = 18; 29 %) and the non-*CHEK2* patients (*n* = 16; 26 %).

**Table 2:** First-line chemotherapy for metastatic breast cancer

	*CHEK2*		Non-*CHEK2*		*P*
	*N*	%	*N*	%	
Type of chemotherapy					
Anthracycline based^a^	34	55	39	63	0.25
Taxane based^b^	6	10	10	16	
Anthracycline/taxane regimen^c^	1	2	1	2	
CMF/CMF-like	21	34	12	19	
Best response					
Objective response	27	44	32	52	0.71
Stable disease	21	35	18	29	
Progressive disease	13	21	12	19	
Unknown	1				
Clinical benefit (objective response and stable disease >6 months)	47	77	48	77	0.96
Progressive disease					
During chemotherapy	26	42	27	44	0.92
After chemotherapy					
No consolidation endocrine therapy	18	29	19	31	
Consolidation endocrine therapy	18	29	16	26	
Anthracycline-based therapy					
Number of patients	34		39		
Best response					
Objective response	18	52	17	43	0.70
Stable disease	8	24	12	31	
Progressive disease	8	24	10	26	
Clinical benefit (objective response and stable disease >6 months)	25	74	27	69	0.69

*CMF* cyclophosphamide, methotrexate and fluorouracil

^a^Anthracycline-based chemotherapy consisted of the following regimens: 19 × FAC, 14 × FEC, 3 × AC in the *CHEK2* group (for three cases, the specific regimen was unknown) and 23 × FAC, 7 × FEC and 4 × AC in the non-*CHEK2* group (*FAC* fluorouracil, adriamycin, cyclophosphamide, *FEC* fluorouracil, epirubicin, cyclophosphamide and *AC* adriamycin, cyclophosphamide)

^b^Taxanes were given 2× in combination with trastuzumab in the *CHEK2* group; and 3× in combination with trastuzumab, 1× in combination with bevacizumab, 1× in combination with trastuzumab and bevacizumab, 2× in combination with methotrexate in the non-*CHEK2* group

^c^Anthracycline/taxane regimen consisted of adriamycin and docetaxel in the *CHEK2* patient and of FAC followed by docetaxel in the non-*CHEK2* patient

The objective response rate to first-line chemotherapy was similar for *CHEK2* and non-*CHEK2* patients (42 and 44 %, respectively), and the clinical benefit rate was also similar in both groups (77 %). In the subgroup analysis of patients receiving an anthracycline-based regimen as first-line chemotherapy for MBC, no significant differences in objective response rate (52 and 43 %, respectively) and clinical benefit rate (74 and 69 %, respectively) were observed between *CHEK2* mutation carriers and non-*CHEK2* patients.

### PFS and OS after start of first-line chemotherapy

**Fig. 2:**
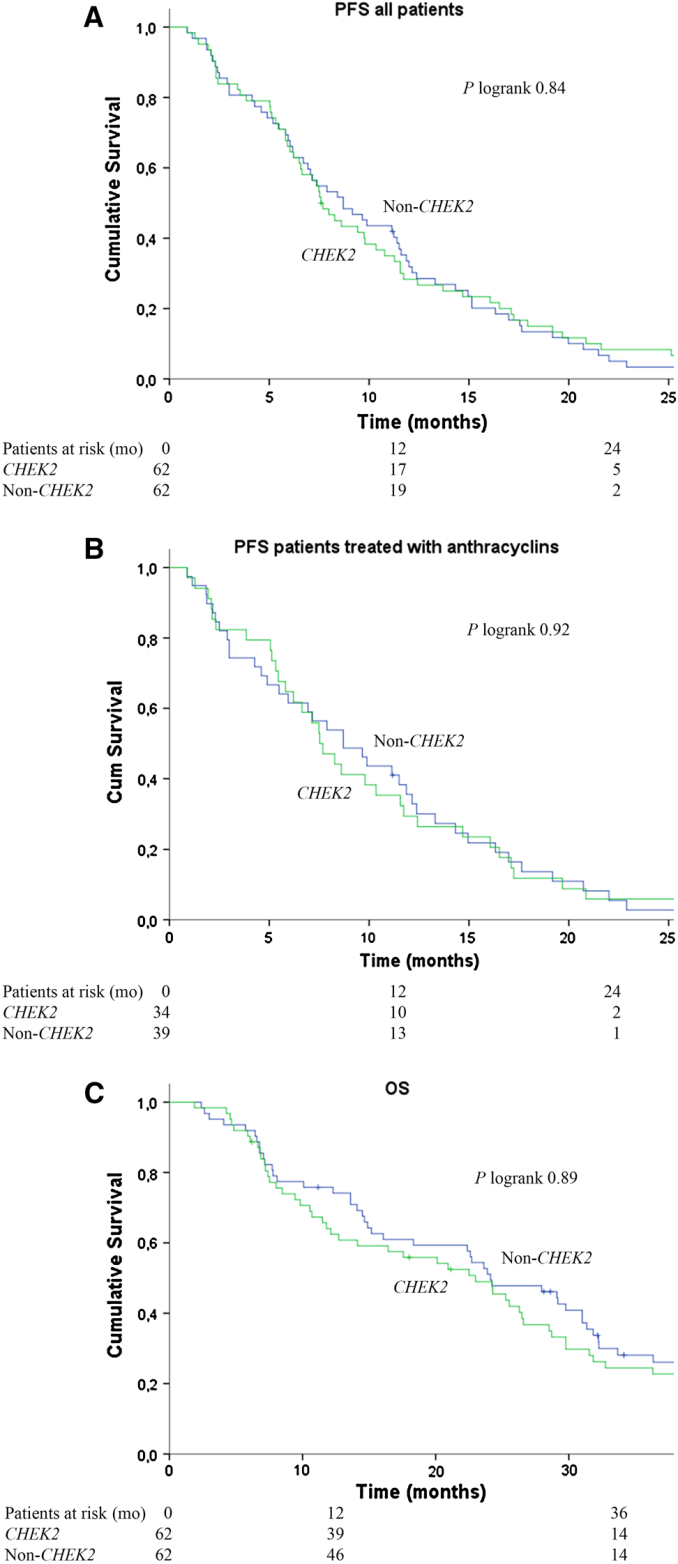
Progression-free survival (PFS) in all metastatic breast cancer patients (**a**) and in metastatic breast cancer patients treated with anthracycline-based chemotherapy (**b**) and overall survival (OS) in metastatic breast cancer patients (**c**)

Data on PFS and OS after start of first-line chemotherapy for MBC are shown in Table 3 and Fig. 2. *CHEK2* and non-*CHEK2* BC patients had a comparable 12-month PFS (28 vs. 32 %) and 12-month OS (64 vs. 76 %). Also, after adjusting for possible confounding factors (see method section), no significant differences in PFS (HR 0.91; 95 % CI 0.63–1.30) and OS (HR 1.03; 95 % CI 0.71–1.49) were observed between the *CHEK2* and non-*CHEK2* groups. As PFS after start of first-line chemotherapy could have been influenced by consolidation endocrine therapy, we also performed the analyses for PFS, with censoring at date of start of consolidation endocrine therapy in respective patients. In these additional analyses, we observed no significantly different PFS between *CHEK2* 1100delC mutation carriers and non-*CHEK2* patients (HR 0.92; 95 % CI 0.63–1.52; data not shown).

**Table 3:** Progression-free and overall survival after first-line chemotherapy

	*CHEK2*		Non-*CHEK2*		*P*
	*N*	%	*N*	%	
Progression-free survival					
*All chemotherapy*					0.84
6 months	41	66	42	68	
12 months	17	28	19	32	
24 months	5	8	2	3	
HR univariate (95 % CI)	0.96 (0.67–1.38)				
HR multivariate (95 % CI)^a^	0.91 (0.63–1.30)				
*Anthracycline*-*based chemotherapy*				0.92	
6 months	22	65	24	62	
12 months	10	29	13	36	
24 months	2	6	1	3	
HR univariate (95 % CI)	0.98 (0.61–1.56)				
HR multivariate (95 % CI)^a^	0.92 (0.57–1.48)				
Overall survival					0.89
6 months	56	90	57	92	
12 months	39	64	46	76	
24 months	28	49	31	51	
36 months	14	24	14	28	
HR univariate (95 % CI)^b^	1.03 (0.71–1.49)				

^a^Adjusted for distant disease-free interval

^b^None of the variables (age at diagnosis, ER status, adjuvant chemotherapy, metachronous contralateral breast cancer, type of first metastases, distant disease-free interval, endocrine therapy for metastatic disease before chemotherapy, time between first distant metastasis and start chemotherapy) had >10 % influence

Further, regarding the analyses for PFS for patients treated with anthracycline-based chemotherapy, no significant differences were observed between CHEK2 and non-*CHEK2* BC patients (at 12 months 29 vs. 36 %, respectively; multivariate HR 0.92; 95 % CI 0.57–1.48).

### First-line endocrine therapy

Response to first-line endocrine therapy for MBC was evaluated in 36 *CHEK2* 1100delC and 32 non-*CHEK2* patients (Table 4). The objective response rate was 14 % in *CHEK2* mutation carriers and 19 % in non-*CHEK2* patients (*P* = 0.82). Also PFS (HR 1.50; 95 % CI 0.90–2.49) and OS (multivariate HR 1.51; 95 % CI 0.89–2.57) after start of first-line endocrine therapy were both not significantly different between *CHEK2* and non-*CHEK2* BC patients.

**Table 4:** First-line endocrine therapy for metastatic breast cancer

	*CHEK2*		Non-*CHEK2*		*P*
	*N*	%	*N*	%	
Number of patients	36		32		
Type of endocrine therapy					
Tamoxifen	26	72	17	53	0.23
Aromatase inhibitor	7	19	9	28	
Other	3	8	6	19	
Best response					
Objective response	5	14	6	19	0.82
Stable disease	18	52	14	45	
Progressive disease	12	34	11	36	
Unknown	1	–	1	–	
Progression-free survival					
6 months	20	56	19	59	0.12
12 months	14	39	13	41	
24 months	3	8	6	19	
HR univariate^a^	1.50	0.90–2.49			
Overall survival					
6 months	36	100	31	97	0.12
12 months	34	94	28	88	
24 months	26	78	26	81	
HR univariate^a^	1.51	0.89–2.57			

^a^None of the added variables (age at diagnosis, ER status, adjuvant chemotherapy, metachronous contralateral breast cancer, type of first metastases, distant disease-free interval) had >10 % influence on the hazard ratio (HR); therefore, no multivariate HR was calculated

## Discussion

Currently, only a few biomarkers are used for therapy stratification in BC patients (e.g., ER, HER2), whereas biomarkers predictive of treatment response will contribute to further tailoring and improving BC treatment, i.e., avoiding toxic therapy for those not benefiting of it. Mutations in BC risk genes might be valuable markers for therapy response prediction, which already has been reported for *BRCA1*- and *BRCA2*-associated BC, suggesting higher sensitivity to anthracyclines, platinum and potentially other agents (Bayraktar and Gluck 2012; Kriege et al. 2009; Tutt et al. 2010).

The current study is the first to address the response to systemic therapy in *CHEK2* 1100delC MBC patients. We observed a similar efficacy of first-line chemotherapy given for MBC in *CHEK2* 1100delC mutation carriers compared to non-*CHEK2* patients. Both response rate to, and PFS and OS after start of first-line chemotherapy were similar in patients with and without the *CHEK2* 1100delC mutation. Also, in patients treated with anthracycline-based chemotherapy, no differential efficacy was observed between *CHEK2* 1100delC-associated and non-*CHEK2* patients. These observations are in line with our previous findings concerning no differential effect of adjuvant chemotherapy on distant disease-free and breast cancer-specific survival in *CHEK2* 1100delC mutation carriers versus non-*CHEK2* patients with early BC (Kriege et al. 2014).

An explanation for the lack of improved efficacy of anthracycline-based chemotherapy in *CHEK2* mutation carriers compared with non-CHEK2 patients, as observed in the current study, might be that a proportion of the BCs diagnosed in *CHEK2* 1100delC mutation carriers are not driven by the loss of CHEK2 function, but still carry a wild-type *CHEK2* allele (either with or without loss of the mutant allele). Finally, the results might be due to the relatively small numbers (34 *CHEK2* and 39 non-*CHEK2* patients treated with anthracyclines).

Our observation regarding no differential efficacy of first-line endocrine therapy for MBC between *CHEK2* 1100delC and non-*CHEK2* patients is in line with the results of our previous study regarding no differential effect of adjuvant endocrine therapy on distant disease-free and BCSS in *CHEK2* and non-*CHEK2* early BC patients (Kriege et al. 2014). To our knowledge, there are no other reports on the efficacy of endocrine therapy in *CHEK2* BC patients yet. In our opinion, however, the question is very relevant as the majority of *CHEK2* BCs is hormone sensitive at diagnosis (de Bock et al. 2004; Kriege et al. 2014; Schmidt et al. 2007; Weischer et al. 2012) and DNA diagnostics for *CHEK2* have recently been implemented in the Netherlands (as part of genetic testing in the context of familial BC) and will be possibly impemented in other countries with a high prevalence. Although we included compared to other series a relative large number of *CHEK2* metastatic breast cancer patients, the number of *CHEK2* patients treated with endocrine therapy for MBC was small (*n* = 36), and future studies with larger sample sizes are needed to address this question further.

Results from the current study in MBC patients and our previous study in early BC patients regarding efficacy of chemotherapy and endocrine therapy do not explain the worse prognosis that has been reported for *CHEK2* 1100delC-associated compared with BC patients without this mutation (de Bock et al. 2004; Kriege et al. 2014; Schmidt et al. 2007; Weischer et al. 2012). Of note, this worse prognosis was not observed in all studies published in CHEK2 mutation carriers to date. A recent paper by Huzarski et al. (Huzarski et al. 2014) did not observe a worse survival in 487 *CHEK2* mutation carriers from Poland; however, in this patient group, the majority of the patients had not the 1100delC, but another CHEK2 variant (Huzarski et al. 2014). Whether there is a difference between patients with these specific *CHEK2* variants in response to (chemo)therapy and survival is unknown yet, although a different response to chemotherapy of different *CHEK2* variants is suggested (Chrisanthar et al. 2008). This deserves further investigation which will require international collaboration as in the Netherlands mainly the *CHEK2* 1100delC mutation is prevalent.

Despite the unique results of the current paper, some limitations should be addressed. Patients included in the current study have been diagnosed and treated in different time periods, resulting in different treatment regimens used. In view of the small number of patients receiving a taxane as first-line chemotherapy for MBC, no conclusions can be drawn for this type of chemotherapy for *CHEK2* 1100delC MBC patients. A second limitation in the analyses regarding the efficacy of chemotherapy is that in some patients, endocrine therapy was given as consolidation therapy after chemotherapy, potentially affecting PFS, but which is common practice in palliative care and outside of clinical trials. The number of patients treated with consolidation endocrine therapy, however, was comparable in the *CHEK2* (*n* = 18) and the non-*CHEK2* groups (*n* = 19). Furthermore, we performed analyses for PFS, with censoring at date of start of consolidation endocrine therapy in respective patients, showing similar results.

DNA diagnostics for hereditary breast cancer is recently extended with the *CHEK2* gene in the Netherlands and in the near future potentially also in other countries having a relatively high prevalence of *CHEK2* mutations. In this view, results of the current study regarding the efficacy of systemic therapy in *CHEK2* 1100delC mutation carriers are very relevant and may helpful with respect to counseling and treatment of respective patients.

In conclusion, the presence of a *CHEK2* 1100delC mutation has currently no impact on the choice of type of systemic treatment in respective MBC patients, neither for chemotherapy nor for endocrine therapy, and our findings do not explain the previously reported worse survival regarding *CHEK2* 1100delC BC. Our observations are important for the counseling and treatment of *CHEK2* 1100delC BC patients, as *CHEK2* 1100delC mutation testing is currently being offered in the Netherlands to familial BC families. On the other hand, the data underscore that further investigations for *CHEK2* 1100delC-associated BC in greater sample sizes, and other specific *CHEK2* mutations, being more prevalent in other countries, are warranted aiming at increased knowledge and further personalized therapy.
